# C-type natriuretic peptide stimulates osteoblastic proliferation and collagen-X expression but suppresses fibroblast growth factor-23 expression in vitro

**DOI:** 10.1186/s12969-020-00441-w

**Published:** 2020-06-09

**Authors:** Wei Xia Chen, Hui Hui Liu, Rui Xue Li, Goshgar Mammadov, Jing Jing Wang, Fei Fei Liu, Sama Samadli, Yang Fang Wu, Dong Dong Zhang, Huang Huang Luo, Peng Hu

**Affiliations:** Department of Pediatrics, The First Affiliated Hospital of Anhui Medical University, No. 218 Ji-Xi Road, Hefei, Anhui Province 230032 PR China

**Keywords:** Bone formation, C-type natriuretic peptide, Fibroblast growth factor-23, Osteoblast, Klotho

## Abstract

**Background:**

The effects of C-type natriuretic peptide (CNP) and fibroblast growth factor (FGF)-23 appear to oppose each other during the process of bone formation, whereas few studies exist on the interaction between CNP and FGF-23. The main objective of the present study is to probe whether CNP is directly responsible for the regulation of osteoblast or via antagonizing FGF-23.

**Methods:**

Osteoblasts were cultured in the absence or presence of CNP (0, 10, and 100 pmol/L) for 24 h, 48 h and 72 h, respectively.

**Results:**

The findings of the present study indicated that: (1) CNP significantly stimulated osteoblastic proliferation and collagen (Col)-X expression; (2) both osteoblastic (osteocalcin, procollagen type I carboxy-terminal propeptide, total alkaline phosphatase and bone-specific alkaline phosphatase) and osteolytic (tartrate-resistant acid phosphatase and cross-linked carboxyterminal telopeptide of type I collagen) bone turnover biomarkers were up-regulated by CNP in osteoblasts; (3) FGF-23 mRNA and protein were significantly down-regulated at 24 h by CNP in osteoblasts, but the expression of FGF receptor-1/Klotho had no significant change.

**Conclusions:**

CNP stimulates osteoblastic proliferation and Col-X expression via the down-regulation of FGF-23 possibly in vitro. However, the specific mechanisms of the interaction between CNP and FGF-23 in osteoblasts are still unclear according to our findings. A further study on osteoblasts cultured with CNP and FGF-23 inhibitor will be undertaken in our laboratory.

## Introduction

C-type natriuretic peptide (CNP) is an endothelium-derived vasodilator and contains two major endogenous forms, designated CNP-53 and CNP-22 [1]. The clearance of CNP-22 in human plasma is very rapid, with a calculated half-life of 2.6 min, shorter than that of CNP-53 [2, 3]. Both of them could result in a significant elevation in intracellular cyclic guanosine monophosphate (cGMP) after selectively binding to the transmembrane natriuretic peptide receptor (NPR)-B [4, 5]. Besides little effect on blood pressure and body fluid homeostasis, CNP mainly elicits local vasorelaxation, inhibits organ remodeling, accelerates reendothelialization, and antagonizes the renin-angiotensin-aldosterone system in a paracrine manner [6–8]. However, several lines of evidence have disclosed that CNP also plays a pivotal role in longitudinal bone growth. Tassano et al. [9] identified the partially overlapping chromosome 2q37 deletions in two patients characterized by short stature and skeletal overgrowth, respectively; the former resulted from the loss of one copy of the CNP precursor gene (*NPPC*) with normal plasma CNP, whereas the latter was affected by overexpression of the *NPPC* and elevation of plasma CNP. Moreover, the heterozygous mutations within the ring structure of CNP could also lead to NPR-B inactivation, cGMP down-regulation and eventually a phenotype of short stature and small hands [10], which is consistent with the previous findings of functional mutation researches on *NPR-B* gene [11, 12]. In *Nppc*^−/−^ mice, the impaired endochondral ossification contributed to early death and severe dwarfism was prevalent in survivals; in contrast, the targeted transgenesis of CNP in the growth plate chondrocytes could rescue the skeletal defect of *Nppc*^−/−^ mice [13]. In addition, a dramatic impairment of endochondral ossification and an attenuation of longitudinal vertebra or limb-bone growth were also found in *Npr-b*^−/−^ mice, whereas the above skeletal abnormalities could be recovered by *Npr-b* overexpression [14]. Therefore, CNP signaling serves as a physiological stimulator of bone growth.

Over the past decades, a few studies in vitro were devoted to the effect of CNP on osteoblasts with a certain controversy. Hagiwara et al. [15] cultured rat osteoblasts with CNP (10^− 7^ M) for 15 days and found that alkaline phosphate (ALP) / osteocalcin transcript and the mineralization of nodules were significantly stimulated with a dose-dependent reduction in the rate of DNA synthesis. However, another study revealed that the constant high ALP activity in mouse osteoblasts was not significantly affected by exogenous CNP (10^− 9^–10^− 5^ M) after a 48-h treatment [16]. Therefore, it should be further elucidated whether the regulatory effect of CNP is a direct consequence on osteoblastic differentiation or mediated by the other cytokines.

Fibroblast growth factor (FGF)-23 is an osteoblast-derived endocrine regulator of phosphate homeostasis through binding to FGF receptor (FGFR)-1 and the co-receptor Klotho, and mainly involved in bone formation [17, 18]. Shimada et al. [19] established an FGF-23 null mouse model and observed that *Fgf-23*^−/−^ mice showed significant hyperphosphatemia, severe growth retardation and short life-span. On the contrary, Larsson et al. [20] generated transgenic mice overexpressing human FGF-23 and found that FGF-23 transgenic mice recapitulated the biochemical and skeletal abnormalities similar to human autosomal dominant hypophosphatemic rickets, oncogenic osteomalacia and X-linked hypophosphatemia. Consistently, Wang et al. [21] transfected human FGF-23 into osteoblasts in vitro and found that overexpressed FGF-23 could significantly suppress osteoblastic differentiation and matrix mineralization. In this context, the main objective of the present study is to probe whether CNP is directly responsible for the regulation of osteoblast or via antagonizing FGF-23 in vitro.

## Materials and methods

### Cell culture

The isolation and culture of osteoblasts were based on the previous study by Declercq et al. [22]. Primary rat calvariae cells (osteoblast-like cells) were isolated from 24 h-old male Sprague Dawley rats, in accordance with the permission of our medical ethics committee (No. LLSC20150009). Briefly, calvariae was dissected and sectioned into fractions. After digestion with 0.1% type II collagenase for 1 h, cells were incubated in α-modified Eagle’s medium (α-MEM; 0.25%; MDL biotech, Beijing, China) supplemented with 12% fetal bovine serum and 1% penicillin/streptomycin at 37 °C in a humidified atmosphere of 95% air and 5% CO_2_. The medium was changed every day and cells from passage 2 to 5 were used in this study. Subsequently, cells were seeded onto 96-well plates at a density of 5.0 × 10^3^/cm^2^ and cultured in α-MEM supplemented with ascorbic acid, β-glycerophosphate and dexamethasone (50 μmol/L, 10 mmol/L and 100 nmol/L, respectively; MDL biotech, Beijing, China) in the absence or presence of CNP-53 (0, 10, and 100 pmol/L; Sangon Biotech, Shanghai, China) for 24 h, 48 h and 72 h, respectively, for its longer half-life than CNP-22.

### Cell proliferation assays

The cell proliferation was measured using the 3-[4,5-dimethylthiazolyl]-2,5-diphenyltetrazolium bromide assay (MTT assay). Briefly, cells were seeded in 96-well plates and cultured in the absence or presence of CNP-53 (0, 10, and 100 pmlo/L) for 24 h, 48 h and 72 h, respectively. Therefore, the cells were then incubated with MTT (MDL biotech, Beijing, China) for another 4 h. Subsequently, the supernatants were removed and the formazan crystals were solubilized in DMSO (MDL biotech, Beijing, China) by constant shaking for 10 min. Finally, optical density (OD) was quantified at 490 nm wavelength using an absorbance reader (ELx800; Bio-tek Inc., Winooski, VT, USA).

### Enzyme-linked immunosorbent assay (ELISA)

The protein levels of bone biomarkers and FGF-23 in the cultured supernatants were determined using commercially available ELISA kits (MDL biotech, Beijing, China) according to the manufacturer’s protocols, including rat osteocalcin (OC) ELISA kit (SEA471Ra), rat procollagen type I carboxy-terminal propeptide (PICP) ELISA kit (SEA570Ra), rat total alkaline phosphatase (tAP) ELISA kit (MD7205), rat bone-specific alkaline phosphatase (bAP) ELISA kit (MD7101), rat tartrate-resistant acid phosphatase (TRAP) ELISA kit (MD7077), cross-linked carboxyterminal telopeptide of type I collagen (ICTP) ELISA kit (MD7064) and rat FGF-23 ELISA kit (MD7106).

### Real-time PCR

Real-time PCR was undertaken with the ABI 7900 sequence detection system (Applied Biosystems, Foster City, CA) using SYBR Green PCR Master Mix (Applied Biosystems, Foster City, CA) in accordance with the manufacturer’s instructions. Total RNA was extracted from cultured osteoblasts by Trizol (ABI-Invitrogen) extraction. Ultraviolet spectrophotometer measuring absorbance and agarose gel electrophoresis confirmed that there had been no degradation of RNA. Among this, 200 ng of isolated RNA were reverse transcribed into cDNA using a SuperScript III RT reagent kit (ABI-Invitrogen). Specific oligonucleotide primers used in the present study were as follows: for FGF-23, sense: 5′-TGG CCA TGT AGA CGG AAC AC-3′, antisense: 5′-GTA GCC GTT CTC TAG CGT CC-3′; for FGFR-1, sense: 5′-CTG GAC CTG AGG CAT CAG TAG-3′, antisense: 5′-AAG CAG CAG CAA TTT TTA TTG AG-3′; for Klotho, sense: 5′-GAC GGG GTT GTA GCC AAG AA-3′, antisense: 5′-CCC AGT CTA GGG AGA ACC GA-3′; for collagen (Col)-X, sense: 5′-TCC CAG GAT TCC CTG GAT CTA A-3′, antisense: 5′-AGG TAT GAC TGC TTG GCT GG-3′; for glyceraldehyde-3-phosphate dehydrogenase (GAPDH), sense: 5′-GTT ACC AGG GCT GCC TTC TC-3′, antisense: 5′-GGG TTT CCC GTT GAT GAC C-3′. Amplification conditions included pre-denaturing at 95 °C for 2 min; then denaturation at 94 °C for 20 s, annealing at 65 °C for 20 s, extension at 72 °C for 30 s; and finally experiencing forty cycles of 30 s at 72 °C. Gene expression was normalized using GAPDH as an endogenous control to correct for differences in the amount of total RNA originally added to each reaction. The average threshold cycle (Ct, the cycles of template amplification to the threshold) was calculated as the value of each sample. Relative quantitative 2^−ΔΔCt^ was used to compare the mRNA expression.

### Immunofluorescence staining

Cells were incubated in 96-well plates and washed three times with cold phosphate buffer saline (PBS) and fixed with 4% paraformaldehyde for 15 min. Subsequently, cells were washed 3 times with PBS and incubated with 0.2% Triton X-100 (Beyotime, Shanghai, China) for 20 min. After washing 3 times in PBS, cells were incubated with 1% bovine serum albumin for 30 min at room temperature. Cells were then incubated overnight at 4 °C with the following antibodies: anti-FGF-23, FGFR-1, Klotho and Col-X (1:50, MDL biotech, Beijing, China). Following this, cells were washed 3 times with PBS and were incubated with secondary antibodies (Donkey-anti-rabbit, Abcam Inc.; Donkey-anti-mouse, Abcam Inc) for 1 h at 37 °C and the nucleus was labeled with 4′,6-diamidino-2-phenylindole (DAPI; 0.1 μg/ml). Finally, the cells were washed with PBS and observed under a Laser scanning confocal microscopy (Nikon A1R, Tokyo, Japan). Representative images were analyzed with Image-Pro Plus 6.0 software, and the mean intensity was calculated.

### Western blot analysis

Cells were cultured in 96-well plates and lysed in cold RIPA buffer (MDL biotech, Beijing, China) with protease inhibitors. Proteins were separated by sodium dodecyl sulfate-polyacrylamide gel electrophoresis and transferred onto polyvinylidene fluoride membranes (Millipore, USA). Membranes were blocked for 1 h at room temperature using 5% fetal bovine serum in tris-buffered saline tween (TBST). Following incubation, membranes were probed with FGF-23, FGFR-1, Klotho, Col-X and actin primary antibodies (MDL biotech, Beijing, China) at 4 °C overnight. After washing 3 times with TBST, membranes were incubated with secondary antibodies for 1 h at room temperature. Finally, membranes were washed 3 times with TBST, and data were captured with a chemiluminescence detection system (Bio-rad, USA).

### Statistical analyses

Statistical analyses were performed using the statistical package for social studies SPSS version 23.0 (SPSS Inc., Chicago, IL). All data were obtained from at least three independent experiments and their values were presented as the mean ± SD. The interaction between treatment and time on bone turnover biomarkers (OC, PICP, tAP, bAP, ICTP and TRAP) were determined using two-way ANOVA. Comparison of mean values among groups was performed using one-way ANOVA, and post hoc analysis was calculated using the Student-Newman-Keuls test. *P* < 0.05 was considered to indicate significance.

## Results

### CNP promoted the proliferation of osteoblasts

**Fig. 1:**
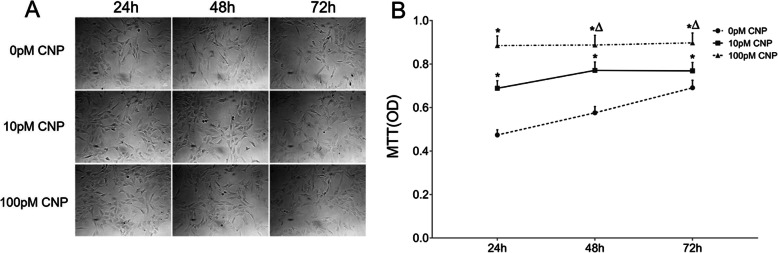
CNP stimulated osteoblast proliferation. Representative microscope images of cells. Original magnification, × 100. The scale bar is 80 μm. The growth of osteoblasts assessed using MTT assay. Data are presented as the mean ± SD. ^*^P < 0.05, significantly different from the corresponding control group (0 pM CNP); ^∆^*P* < 0.05, significantly different from the corresponding low-dose group (10 pM CNP)

Under microscope, active osteoblasts were plump, cuboidal, mononuclear cells lying on the matrix which they synthesized (Fig. 1a). The evaluation of osteoblastic proliferation is presented in Fig. 1b. Based on the result of MTT assays, CNP significantly promoted the proliferation of osteoblasts in a dose-dependent manner. More specifically, the baseline data of OD were 0.47 ± 0.024, 0.58 ± 0.029, and 0.69 ± 0.035 at 24 h, 48 h, and 72 h in cultured cells without CNP treatment respectively. The OD value were 0.69 ± 0.034, 0.77 ± 0.039, and 0.77 ± 0.038 in the low-dose group at 24 h, 48 h and 72 h post-treatment respectively, and 0.89 ± 0.044, 0.89 ± 0.044, and 0.90 ± 0.045 in the high-dose group at 24 h, 48 h and 72 h post-treatment respectively. Furthermore, the high-dose group showed a 0.28-, 0.15- and 0.17-fold increase compared with the corresponding low-dose group at 24 h, 48 h and 72 h post-treatment respectively (*P* < 0.05).

### The effects of CNP on bone turnover biomarkers

**Fig. 2:**
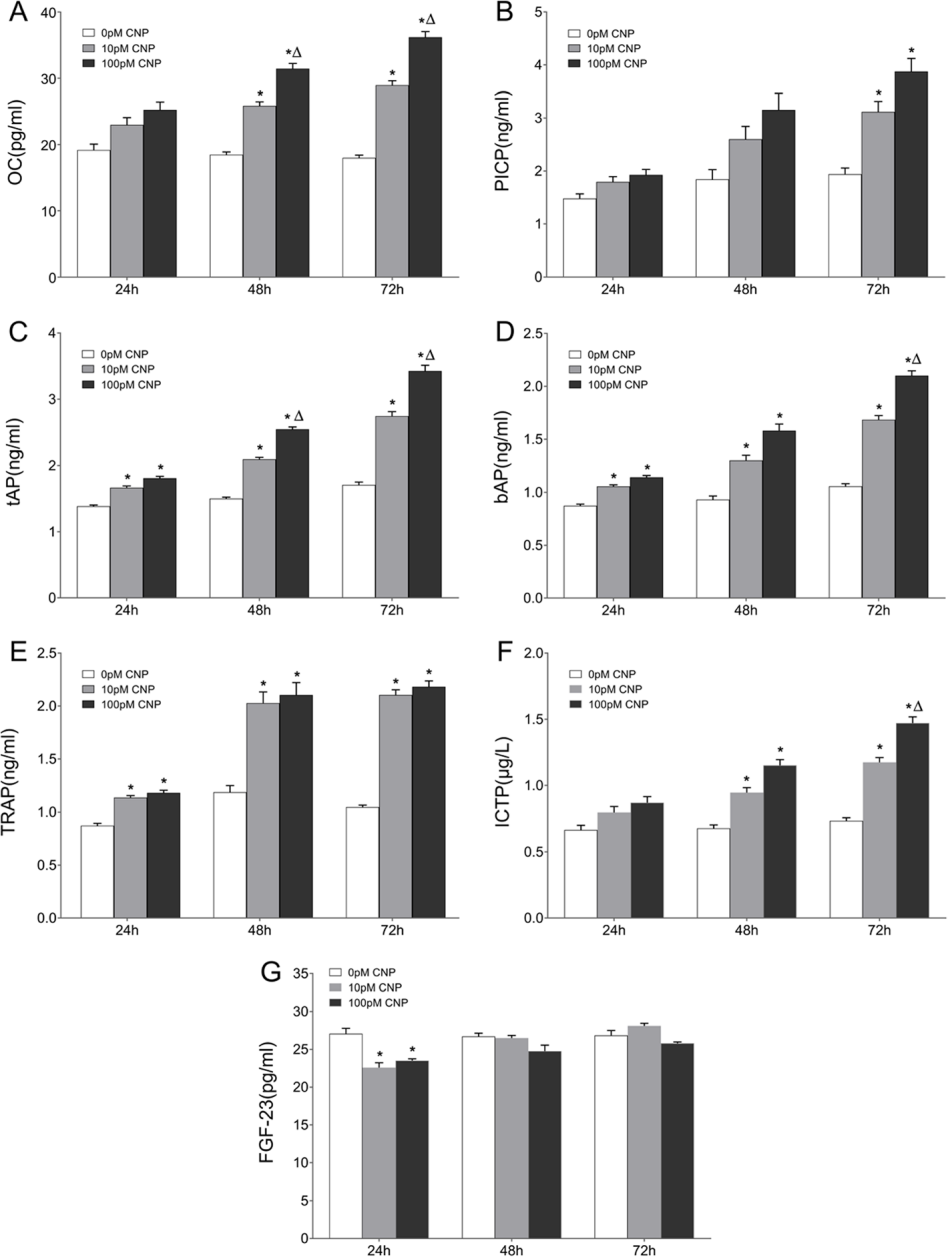
ELISA analysis of OC, PICP, tAP, bAP, TRAP and ICTP in the cultured osteoblasts. The supernatant levels of (**a**) OC, (**b**) PICP, (**c**) tAP, (**d**) bAP, (**e**) TRAP and (**f**) ICTP were determined using commercially available ELISA kits after CNP treatment (96-well plates). Data are presented as the mean ± SD. ^*^P < 0.05, significantly different from the corresponding control group (0 pM CNP); ^∆^*P* < 0.05, significantly different from the corresponding low-dose group (10 pM CNP)

Figure 2 illustrates the supernatant levels of osteoblastic (OC, PICP, tAP, bAP) and osteolytic (TRAP and ICTP) biomarkers by ELISA. Compared with the corresponding controls, supernatant OC displayed a marked increment in both the low-dose group and high-dose group at 48 h and 72 h post-treatment, respectively (a 0.40- and 0.70-fold increase at 48 h; a 0.61- and 1.01-fold increase at 72 h, *P* < 0.05); furthermore, supernatant OC experienced a 0.22- and 0.25-fold increase in the high-dose group at 48 h and 72 h post-treatment, compared with their lose-dose counterparts, respectively (Fig. 2a, *P* < 0.05). In comparison with the control group, supernatant PICP showed a significant elevation in both the low-dose group (a 0.61-fold increase) and high-dose group (a 1.00-fold increase) only at 72 h post-treatment, respectively (Fig. 2b, *P* < 0.05). In comparison with the corresponding controls, significant higher levels of tAP and bAP were noted in the CNP-treated groups at each time point. Supernatant tAP showed a 0.32-, 0.40- and 0.61-fold increase in the low-dose group at 24 h, 48 h and 72 h post-treatment respectively, and a 0.43-, 0.70- and 1.00-fold increase in the high-dose group at 24 h, 48 h and 72 h post-treatment respectively (Fig. 2c, *P* < 0.05). Supernatant bAP showed 0.21-, 0.40- and 0.59-fold increase in the low-dose group at 24 h, 48 h and 72 h post-treatment respectively, and a 0.31-, 0.70- and 0.99-fold increase in the high-dose group at 24 h, 48 h and 72 h post-treatment respectively (Fig. 2d, *P* < 0.05). In addition, compared with the corresponding low-dose group, supernatant tAP presented an apparent increase in the high-dose group at 48 h (a 0.21-fold increase) and 72 h (a 0.25-fold increase) post-treatment (Fig. 2c, *P* < 0.05), whereas the significant elevation of bAP was observed in the high-dose group only at 72 h post-treatment (a 0.25-fold increase, Fig. 2d, *P* < 0.05). In addition, compared with the corresponding low-dose group, supernatant tAP presented an apparent increase in the high-dose group at 48 h (a 0.21-fold increase) and 72 h (a 0.25-fold increase) post-treatment (Fig. 2c, *P* < 0.05), whereas the significant elevation of bAP was observed in the high-dose group only at 72 h post-treatment (a 0.25-fold increase, Fig. 2d, *P* < 0.05). In comparison with the corresponding controls, supernatant TRAP experienced an obvious increase in the CNP-treated groups at each time point (a 0.30-, 0.71- and 1.01-fold increase in the low-dose group at 24 h, 48 h and 72 h respectively; a 0.35-, 0.77- and 1.09-fold increase in the high-dose group at 24 h, 48 h and 72 h, respectively, Fig. 2e, *P* < 0.05). Compared with the corresponding controls, supernatant ICTP were also significantly upregulated in the CNP-treated groups at 48 h and 72 h (a 0.40- and 0.60-fold increase in the low-dose group at 48 h and 72 h, respectively; a 0.70- and 1.00-fold increase in the high-dose group at 48 h and 72 h, respectively, Fig. 2f, *P* < 0.05); in addition, supernatant ICTP exhibited a 0.25-fold increase in the high-dose group at 72 h post-treatment in comparison with the corresponding low-dose counterpart (Fig. 2f, *P* < 0.05). Subsequently, two-way ANOVA analysis revealed that CNP treatment and time had significant effects on all the bone turnover biomarkers (*F* ≥ 5.91, *P* < 0.05).

### CNP upregulated col-X

**Fig. 3:**
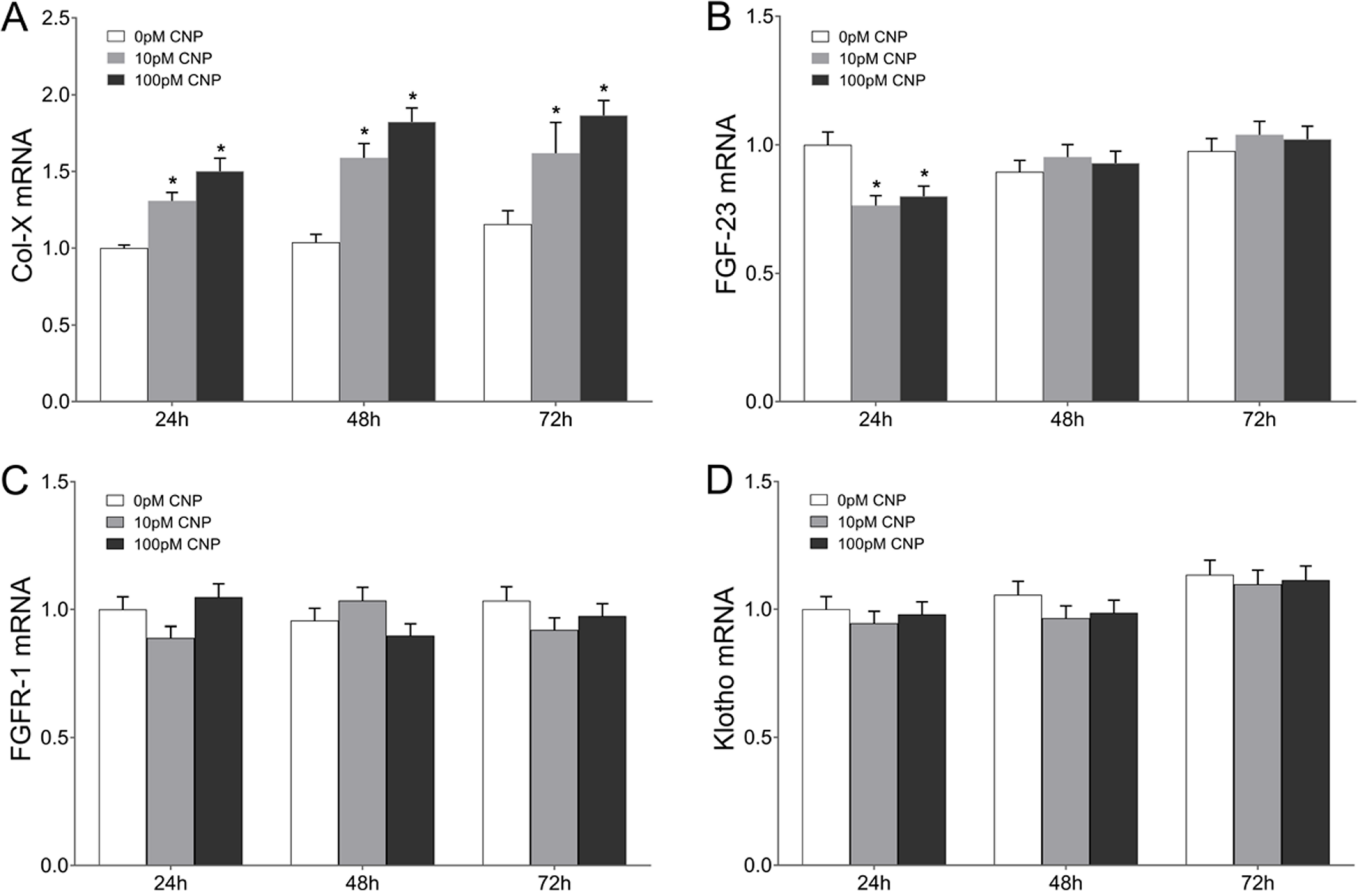
Real-time PCR analysis of Col-X, FGF-23, FGFR-1, and Klotho mRNA in osteoblasts. **a** Col-X, **b** FGF-23, **c** FGFR-1, and **d** Klotho mRNA expression in osteoblasts were measured by real-time PCR (96-well plates). Data are presented as the mean ± SD. ^*^P < 0.05, significantly different from the corresponding control group (0 pM CNP); ^∆^*P* < 0.05, significantly different from the corresponding low-dose group (10 pM CNP)

**Fig. 4:**
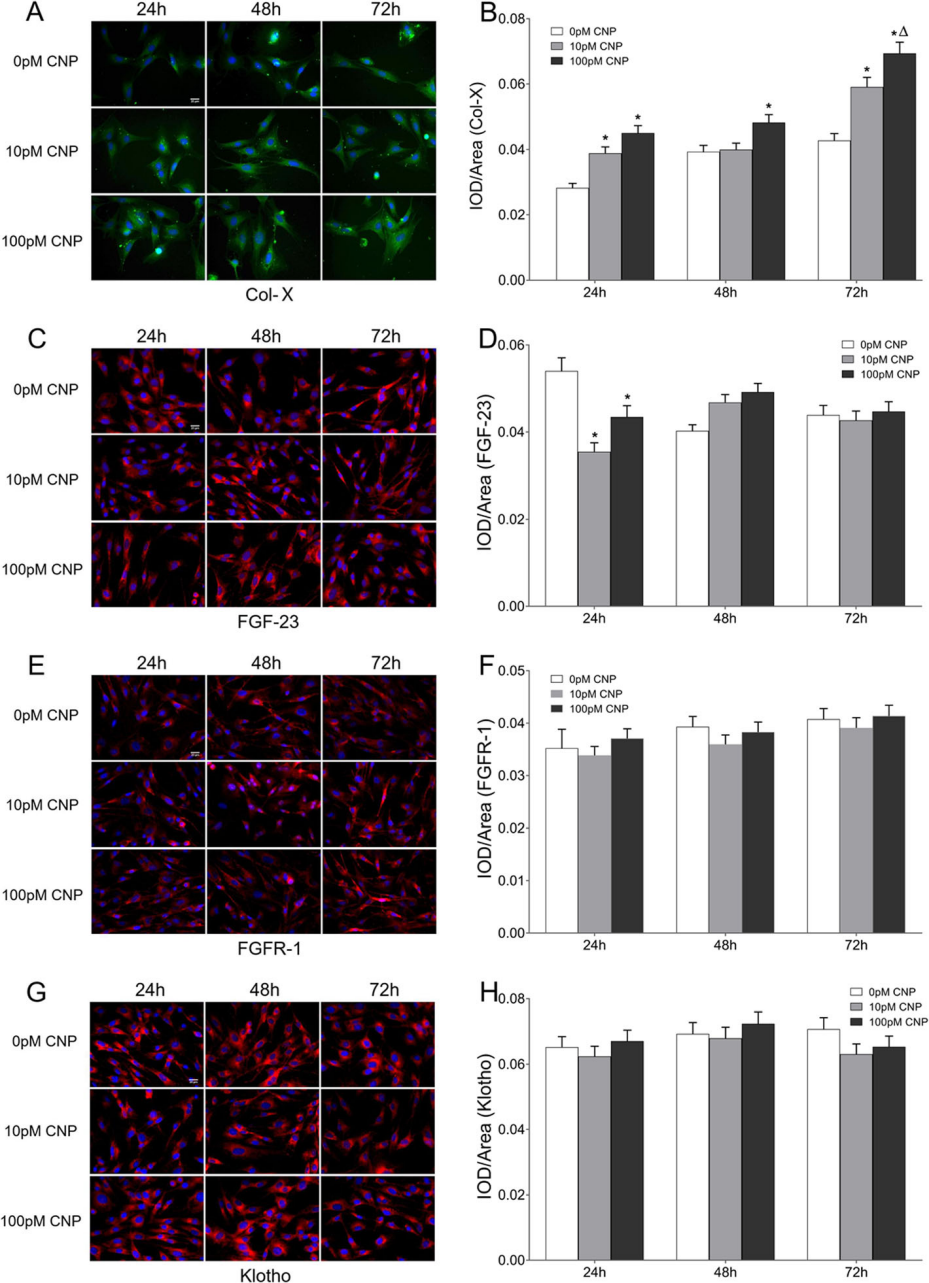
Immunofluorescence staining of Col-X, FGF-23, FGFR-1 and Klotho protein in osteoblasts. Representative microscope images of cells on the coverslips with DAPI-stained nuclei (blue) and Col-X staining (green)/FGF-23 staining (red)/FGFR-1staining (red)/Klotho staining (red). Original magnification, × 400. The scale bar is 20 μm. **a** Col-X, **c** FGF-23, **e** FGFR-1 and **g** Klotho. Fluorescence intensities of Col-X, FGF-23, FGFR-1 and Klotho. **b** Col-X, **d** FGF-23, **f** FGFR-1 and **h** Klotho. Data are presented as the mean ± SD. ^*^P < 0.05, significantly different from the corresponding control group (0 pM CNP); ^∆^*P* < 0.05, significantly different from the corresponding low-dose group (10 pM CNP)

Real-time PCR analysis of Col-X mRNA is shown in Fig. 3. Compared with the corresponding control group, Col-X mRNA expression exhibited a 0.31-, 0.53- and 0.40-fold increase in the low-dose group at 24 h, 48 h and 72 h post-treatment respectively, and a 0.50-, 0.76- and 0.61-fold increase in the high-dose group at 24 h, 48 h and 72 h post-treatment, respectively (Fig. 3a, *P* < 0.05). Subsequently, the expression characterization of Col-X was confirmed using Western blot (data not shown). Compared with the corresponding control group, the protein expression of Col-X experienced a 0.27-, 0.24- and 0.30-fold increase in the low-dose group at 24 h, 48 h and 72 h post-treatment respectively, and a 0.39-, 0.39- and 0.64-fold increase in the high-dose group at 24 h, 48 h and 72 h post-treatment respectively (*P* < 0.05). In addition, the high-dose group showed a 0.26-fold increase of Col-X protein than that in the low-dose group at 72 h post-treatment (*P* < 0.05). To clarify the distributions of Col-X protein in osteoblasts, immunofluorescence staining was performed. As shown in Fig. 4a, Col-X positive reaction was noted in both the control and CNP-treated group throughout the whole observational period, but more significant in the CNP-treated group than controls. More specifically, semi-quantitative analysis indicated that the cumulative optical density per unit area (IOD/area) was significantly increased in the high-dose group at each time point, and in the low-dose group at 24 h and 72 h post-treatment, compared with the corresponding controls. Moreover, the IOD/area was greater in the high-dose group than that in the low-dose group only at 72 h post-treatment (Fig. 4b, *P* < 0.05).

### CNP inhibited the expression of FGF-23 in the earlier phase

Figure 2g depicts the supernatant levels of FGF-23 by ELISA. Supernatant FGF-23 experienced an 18% decrease in the low-dose group and a 13% decrease in the high-dose group only at 24 h post-treatment, compared with the baseline of controls (*P* < 0.05). In osteoblasts, the relative quantity of FGF-23 transcript exhibited a 24% decrease in the low-dose group and a 20% decrease in the high-dose group only at 24 h post-treatment (Fig. 3b, *P* < 0.05), which was confirmed using Western blot (data not shown). In addition, immunofluorescence staining showed that the osteoblasts were positive for FGF-23 throughout the observational period, whereas its IOD/area also presented a marked decrease at 24 h after CNP treatment (Fig. 4c, d, *P* < 0.05). Therefore, CNP inhibited the expression of osteoblastic FGF-23 in the earlier phase.

### No effect of CNP on FGFR-1/Klotho

By real-time PCR analysis, no significant differences in FGFR-1/Klotho expression were found between the control group and the CNP-treated group at each time point (Fig. 3, *P* > 0.05), which was confirmed using Western blot (data not shown). FGFR-1/Klotho expression also showed a dose-independent manner after CNP treatment (Fig. 3c, d, *P* < 0.05). In addition, immunofluorescence staining showed that osteoblasts were positive for FGFR-1/Klotho staining in both the control and CNP-treated group throughout the whole observational period (Fig. 4e, Fig. 4g). Evaluation of IOD/area revealed no significant differences between the control group and the CNP-treated group at each time point, and the IOD/area was not in parallel with the dose of CNP (Fig. 4f, h, *P* < 0.05). Therefore, CNP did not affect the expression of FGFR-1/Klotho.

## Discussion

Osteoblasts mediate the process of mineralization by producing ALP and secreting matrix vesicles to facilitate hydroxyapatite crystal formation, and this process is regulated by various hormones such as growth hormone, insulin-like growth factor, parathyroid hormone and 1,25-dihydroxyvitamin D3 [23, 24]. Over the past two decades, increasing evidence has demonstrated CNP importance as a physiological stimulator of longitudinal bone growth, chondrocytic proliferation and hypertrophy, and cartilage matrix synthesis [9, 25]. Pathologically, our latest study established a rat model of renal osteodystrophy and observed that CNP administration significantly restored calcium phosphate metabolic disorders, hypovitaminosis D, secondary hyperparathyroidism and decreased bone turnover markers, and retarded bone pathological progression [26]. To eliminate disturbance from the neuroendocrine network, the present study cultured osteoblasts with different doses of CNP and found that osteoblastic proliferation could be directly promoted by exogenous CNP in a dose-dependent manner in vitro. However, the proliferative regulation of CNP shows significant diversity among different cell types. Two previous studies from Khambata’s and our laboratory revealed that CNP caused a concentration-dependent inhibition of cell proliferation in vascular smooth muscle cells and renal mesangial cells, but led to a concentration-dependent augmentation of cell growth in vascular endothelial cells [8, 27]. Although the potential mechanism is still unclear to date, further studies on cell cycle regulation may provide more insight into the different proliferative regulations of CNP on different cells.

Bone turnover biomarkers reflect the metabolic status of the entire skeleton. There are two groups of bone turnover biomarkers: osteoblastic (OC, PICP, tAP and bAP) and osteolytic (ICTP and TRAP) biomarkers [28]. In the present study, both osteoblastic and osteolytic biomarkers were significantly up-regulated in osteoblasts cultured with CNP; more specifically, the larger increase in osteoblastic biomarkers than osteolytic biomarkers may be subject to bone formation. Similarly, Hagiwara et al. [15] cultured osteoblasts with CNP (10^− 7^ M) and found that ALP activity became ~ 140% of the base level accompanied by 2-fold increase of ALP transcript on the 9th day, and OC transcript was obviously promoted on the 15th day. However, the osteolytic biomarkers were not observed in the above study. Kondo et al. [29] investigated the bone phenotype of a mouse model with elevated plasma CNP concentrations (SAP-CNP-Tg mice) and found that 8-week old SAP-CNP-Tg mice showed enhanced osteoblastic and osteolytic activities, in accordance with elevated serum levels of OC and TRAP. Therefore, CNP is considered as a positive regulator of bone formation both in vitro and in vivo.

Col-X is a short-chain non-fibrillar collagen deposited at sites of new bone formation, and facilitates endochondral ossification by regulating matrix mineralization and compartmentalizing matrix components [30, 31]. Several signaling molecules have been implicated in regulation of Col-X metabolism. These include the positive regulators (thyroid hormones, retinoic acid, bone morphogenetic proteins and transcription factor Runx2), as well as the negative regulator (Indian hedgehog and parathyroid hormone-related protein) [32]. Although CNP is considered as a positive regulator of bone formation, limited evidence exists on the accelerative effect of CNP on Col-X during the process of bone formation. Miyazawa et al. [33] conducted an organ culture study to observed Col-X expression of mouse tibias after CNP (10^− 7^ M) treatment and found an appreciable increase in the extracellular space positive for Col-X on the 4th day post-culture. In addition, the accelerative effect of CNP on Col-X transcript was also identified in the micromass culture of the chick limb mesenchymal cells and chondrocytes in vitro [34, 35]. Conformably, in the present study, CNP dramatically promoted osteoblastic Col-X expression in both mRNA and protein levels in vitro. However, whether the regulative effect of CNP is a direct consequence on Col-X or mediated by the other cytokines should be further elucidated.

FGF-23 is produced by osteoblasts and acts on the kidneys and parathyroid glands to maintain phosphate homeostasis and regulate vitamin D synthesis in an endocrine manner [36]. Recent studies of human genetic disorders and genetically engineered mice, as well as the in vitro approaches, have proved that FGF-23 overexpression not only suppresses osteoblastic proliferation but also impairs bone mineralization [37, 38]. In a transgenic mouse model overexpressing wild-type human FGF-23, tibial pathology displayed a disorganized and widened growth plate, reduced bone mineral density and impaired mineralization [20]. Teerapornpuntakit et al. [39] cultured rat osteoblast-like UMR-106 cells with FGF-23 (100 ng/mL), and noted that cell proliferation was significantly down-regulated by FGF-23 at 48 h. Besides the suppressed osteoblastic proliferation and impaired bone mineralization, FGF-23 can also disturb Col-X metabolism. Wu et al. [40] transfected human FGF-23 into rat mandibular cartilage chondrocytes and observed that the overexpressed FGF-23 significantly inhibited the expression of Col-X transcript in vitro. In our latest study, we established a rat model of renal osteodystrophy and found that serum levels and bone expression of FGF-23 were both significantly elevated in uremic rats; and moreover, serum FGF-23 was negatively correlated with bone Col-X [41]. Currently, many systemic and local factors have been identified to participate in FGF-23 regulation, including calcitriol, phosphate, parathyroid hormone, calcium, and so on [42, 43]. To the best of our knowledge, the effects of CNP and FGF-23 appear to be opposite during the process of bone formation, whereas few studies exist on the interaction between CNP and FGF-23. The present study suggested that CNP treatment inhibited osteoblastic FGF-23 expression in the earlier phase combined with elevated Col-X expression in vitro. Given this background, we hypothesize that CNP may stimulate Col-X expression via the down-regulation of FGF-23. Furthermore, compared with the controls, supernatant OC, tAP and bAP displayed a marked increment in both the low-dose group and high-dose group at 48 h and 72 h post-treatment, respectively, whereas the changes in FGF-23 were not significant in both groups at 48 h and 72 h treated by CNP. On the other hand, the high-dose group showed a significant increase of Col-X protein compared with in the low-dose group at 72 h post-treatment, whereas the expression of Col-X at 24 h and 48 h was similar in both groups after treated by CNP. Based on these findings, we speculate that this may be due to a shorter half-life of CNP and the hysteresis effects of FGF-23.

FGF-23-FGFR-1 complex plays regulative roles in bone development and disease [44]. Wang et al. [21] transfected human FGF-23 into osteoblasts in vitro and found that overexpressed FGF-23 obviously suppressed nodule formation and mineralization accompanied by enhanced phosphorylation of FGFR-1. On the contrary, in a mouse model of X-linked hypophosphatemia, Xiao et al. [45] found that the conditional deletion of *Fgfr-1* in osteocytes contributed to a 30% reduction in bone FGF-23 expression and a 70% reduction in serum FGF-23 concentration as well as a significant improvement in rickets and osteomalacia. On the other hand, binding of FGF-23 to the canonical FGFR-1 requires the obligatory co-receptor Klotho [46]. Shalhoub et al. [38] cultured osteoblasts with FGF-23 in the absence or presence of soluble Klotho, and noted that FGF-23 plus Klotho led to inhibition of mineralization and osteoblast activity markers on day 14; in contrast, neither FGF-23 nor Klotho exposure alone affected proliferation of day 4 growth phase cells or mineralization of day 14 cultures. However, the effects of CNP on FGFR-1/Klotho are seldom reported in osteoblasts to data. In the present study, FGF-23 mRNA and protein were significantly down-regulated by CNP in osteoblasts, but the expression of FGFR-1/Klotho had no significant change. Thus, FGF-23 may elicit its effects in a FGFR-1/Klotho-independent fashion in osteoblast.

FGF-23/ mitogen-activated protein kinase (MAPK) signaling pathway is known to be suppressing osteoblastic activity and this is via MAPK activation [38]. However, the crosstalk between CNP and FGF-23/MAPK signaling has not been extensively studied so far. Yasoda et al. [47] treated tibial explants with CNP (10^− 6^ and 10^− 7^ M) or its second messenger, cGMP (10^− 5^ M), before addition of FGF-2 (2 ng/ml, similar to FGF-23) and found that FGF-2-induced phosphorylation of extracellular regulated protein kinase (ERK) 1/2 was markedly decreased in a dose-dependent fashion. Moreover, identical results were obtained using the chondrogenic cell line ATDC5. In our previous study, we established a renal osteodystrophy rat model to identify whether CNP could attenuate renal osteodystrophy through the inhibition of FGF-23 cascades, and found that a continuous infusion of CNP (0.05 μg/kg/min × 1 h) significantly inhibited the expression of FGF-23, RAF-1/phospho-RAF-1, and downstream ERK/phospho-ERK in bone tissue [26]. As for FGF-23 signaling, no dose effect of CNP was observed in the present study. The expression of FGF-23 were significantly suppressed in both low-dose (10 pmol/L) and high-dose (100 pmol/L) groups at 24 h post-treatment. Based on the transcription and protein levels of FGF-23, there was a trend toward low-dose group to experience a more obvious decrease. A further research should be undertaken to observe the potential mechanism.

## Conclusions

In summary, our study revealed, for the first time, that exogenous CNP stimulated osteoblastic proliferation and Col-X expression via the down-regulation of FGF-23 possibly in vitro. However, FGFR-1 and Klotho were not influenced by CNP in osteoblasts. According to the findings of present study, the specific mechanism of the interaction between CNP and FGF-23 in osteoblasts is still unclear. Currently, the further study on osteoblasts cultured with CNP and FGF-23 inhibitor is being taken in our laboratory.

## Data Availability

The datasets generated and/or analyzed during current study are available from the corresponding author on reasonable request.

## Abbreviations

ALP: Alkaline phosphatase
bAP: Bone-specific alkaline phosphatase
cGMP: Cyclic guanosine monophosphate
CNP: C-type natriuretic peptide
Col-X: Collagen X
ICTP: Cross-linked carboxyterminal telopeptide of type I collagen
ELISA: Enzyme-linked immunosorbent assay
ERK: Extracellular regulated protein kinase
FGF-23: Fibroblast growth factor-23
FGFR-1: Fibroblast growth factor receptor-1
GAPDH: Glyceraldehyde-3-phosphate dehydrogenase
MAPK: Mitogen-activated protein kinase
NPR-B: Natriuretic peptide receptor-B
OD: Optical density
OC: Osteocalcin
PBS: Phosphate buffer saline
PICP: Procollagen type I carboxy-terminal propeptide
tAP: Total alkaline phosphatase
TBST: Tris-buffered saline tween
TRAP: Tartrate-resistant acid phosphatase
IOD/area: The cumulative optical density per unit area
MTT assay: 3-[4,5-dimethylthiazolyl]-2,5-diphenyltetrazolium bromide assay
